# Nanoscale twinning in Fe–Mn–Al–Ni martensite: a backscatter Kikuchi diffraction study

**DOI:** 10.1107/S1600576720013631

**Published:** 2021-02-01

**Authors:** Peter D. B. Fischer, Stefan Martin, Alexander Walnsch, Martin Thümmler, Mario J. Kriegel, Andreas Leineweber

**Affiliations:** a Institute of Materials Science, TU Bergakademie Freiberg, Gustav-Zeuner-Strasse 5, Freiberg, 09599, Germany

**Keywords:** electron backscatter diffraction, martensite, shape-memory alloys, twinning, backscatter Kikuchi diffraction

## Abstract

Iron-based Fe–Mn–Al–Ni shape-memory alloys are of rather low materials cost and show remarkable pseudoelastic properties. To further understand the martensitic transformation giving rise to the pseudoelastic properties, different Fe–Mn–Al–Ni alloys have been heat treated at 1473 K and quenched in ice water. The martensite, which is formed from a body-centred cubic austenite, is commonly described as face-centered cubic (f.c.c.), even though there are also more complex, polytypical descriptions of martensite. The presently studied backscatter Kikuchi diffraction (BKD) patterns have been evaluated, showing a structure more complex than simple f.c.c. This structure can be described by nanoscale twins, diffracting simultaneously in the exciting volume. The twinned structure shows a tetragonal distortion, not uncommon for martensite in spite of the lack of interstitial elements. These features are evaluated by comparing the measured BKD patterns with dynamically simulated ones.

## Abstract 2

Fe–Mn–Al–Ni martensite distortion gives rise to complex backscattered Kikuchi diffraction patterns which cannot be interpreted by standard procedures. Analysis of these patterns reveals that they arise from nanoscale internal twinning and tetragonal distortion of the basically cubic close-packed martensite.

## 1. Introduction   

Iron-based Fe–Mn–Al–Ni shape-memory alloys (SMAs) show a remarkable pseudoelastic hysteresis (Vollmer *et al.*, 2015 ▸, 2016 ▸, 2017 ▸, 2019 ▸; Omori *et al.*, 2011 ▸, 2012 ▸, 2013 ▸) and are, therefore, of special interest. Moreover, the low costs of the alloying elements and the production of Fe-based SMAs as compared with other SMAs, *e.g.* Ni–Ti-based SMAs, make this materials system promising for future applications (Omori *et al.*, 2013 ▸). To further improve the mechanical properties, a fundamental understanding of the martensitic transformation giving rise to the shape-memory effect in this system is essential.

Quite consistently, the parent austenite phase in Fe–Mn–Al–Ni SMAs is described as a body-centered cubic (b.c.c.) crystal structure. The martensite is usually described with a face-centered cubic (f.c.c.) structure (Omori *et al.*, 2011 ▸, 2012 ▸, 2013 ▸; Tseng *et al.*, 2015*a* ▸,*b* ▸, 2016*a* ▸,*b* ▸; Vallejos *et al.*, 2018 ▸; La Roca *et al.*, 2015 ▸). Omori *et al.* (2012 ▸) have reported, on the basis of selected-area electron diffraction of the martensite, for the most prominent alloy composition Fe_43.5_Mn_34_Al_15_Ni_7.5_ (the numbers indicate at.%) that the f.c.c. martensite is (on average) actually a long-range stacking faulted 53 polytype^1^ (Zhdanov notation) (Zhdanov, 1945 ▸) referred to as nano­twinned. Taking a tentative f.c.c. lattice parameter of *a*_fcc_ = 3.6 Å, the twin boundaries perpendicular to the stacking direction 〈111〉 in this structure can be calculated to have distances of only 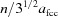 with *n* = 3 and 5 (*i.e.* 6.2 and 10.4 Å). Due to the very small sizes of twin lamellae and the distinct distance between them, the depicted electron diffraction patterns differ considerably from those of independently diffracting f.c.c. twins. A later backscatter Kikuchi diffraction study shows maps with large regions (of a size of several µm) of uniformly indexed martensite in Fe_43.5_Mn_34_Al_15_Ni_7.5_ alloys (Vallejos *et al.*, 2018 ▸), where according to the authors an f.c.c. structure had been used for indexing. However, no reference has been made to the previous result of a far more complicated atomic structure of martensite reported by Omori *et al.* (2012 ▸).

**Figure 1:**
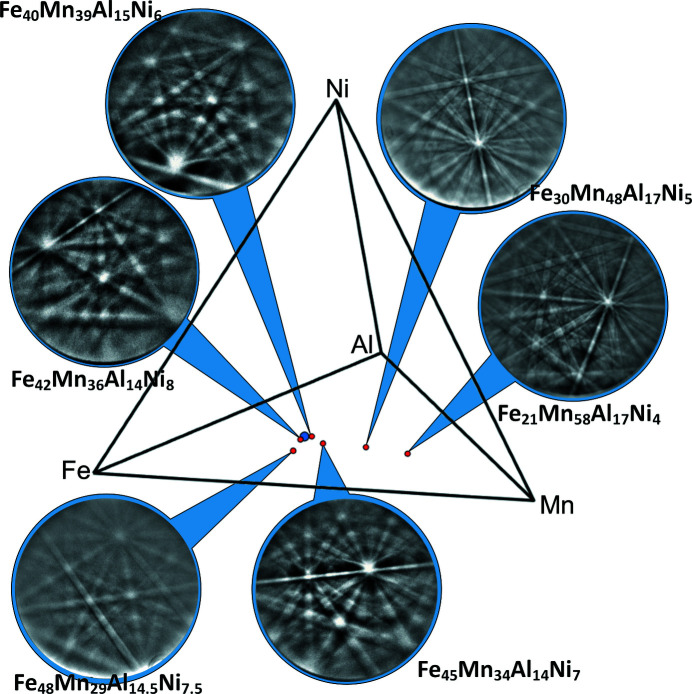
Tetrahedral plot depicting the mole fractions of a series Fe–Mn–Al–Ni alloys containing f.c.c.-type martensite which formed from b.c.c. parent austenite (red dots). Additionally the composition of the frequently employed Fe_43.5_Mn_34_Al_15_Ni_7.5_ is highlighted with a blue dot. For each investigated composition a backscatter Kikuchi diffraction pattern of the martensite is shown, revealing higher diffraction pattern quality for alloys with higher Mn content.

In the course of our own investigations on Fe–Mn–Al–Ni alloys (Walnsch *et al.*, 2019*a* ▸), even in the case of quite reasonable quality of the backscatter Kikuchi diffraction (BKD) patterns originating from martensite, we encountered difficulties during indexing when relying on an f.c.c. structure with Hough-space-based routines. Hence, we attempted to reveal the characteristics of the patterns which obstruct indexing. In Fig. 1 ▸ BKD patterns recorded for martensite in a series of alloys with about 15 at.% Al and 7.5 at.% Ni are shown. The Kikuchi band edges were found to be sharper for the alloys with higher Mn contents, suggesting a lower degree of inhomogeneous strain in these alloys. Therefore, it was decided to perform the current study based on the martensite in an Mn-rich Fe_21_Mn_58_Al_17_Ni_4_ alloy instead of the usually considered Fe_43.5_Mn_34_Al_15_Ni_7.5_ alloy. However, the features characterized within the present work are also relevant for the martensite of other compositions (see Fig. 1 ▸), *e.g.* close to the chemical composition of the pseudoelastic alloy usually considered.

This study will mainly focus on the acquisition of structural information obtained from BKD patterns. The effect of the crystal structure and microstructure on the martensite in this alloying system will be described elsewhere.

## 2. Experimental   

An Fe_21_Mn_58_Al_17_Ni_4_ alloy of 3 g was prepared from the pure metals by arc melting. The resulting ingot (dimensions: cylinder with 1.5 cm diameter and 5 mm height) was afterwards heat-treated at 1473 K in an argon atmosphere for 24 h and quenched in ice water. The chemical compositions were checked by energy-dispersive X-ray spectroscopy (EDS). Further details regarding the sample preparation and annealing procedures can be found in the work of Walnsch *et al.* (2019*a* ▸,*b* ▸). Thermally induced martensite was generated by water quenching from the heat-treatment temperature.

Scanning electron microscopy (SEM) on metallographically prepared cross sections (special effort has been made to prevent re-heating of the samples during the preparation process) after grinding and polishing (final stage: vibrational polishing) has been used to acquire the shown figures and BKD patterns. The austenite grains have a size of several hundred µm, making powder X-ray diffraction an unsuitable method for investigating the crystal structure and microstructure. Therefore, electron backscatter diffraction (EBSD) was chosen as the investigation method.

EBSD was carried out using a JEOL JSM 7800F scanning electron microscope with an acceleration voltage of 30 keV and beam current of approximately 10 nA. The BKD camera (EDAX Hikari Super Elite) has a resolution of 480 × 480 pixels and the output patterns are in a 16-bit format, making a precise analysis of the diffracted intensities possible. The BKD patterns were acquired using the EDAX *TEAM* software.

**Figure 2:**
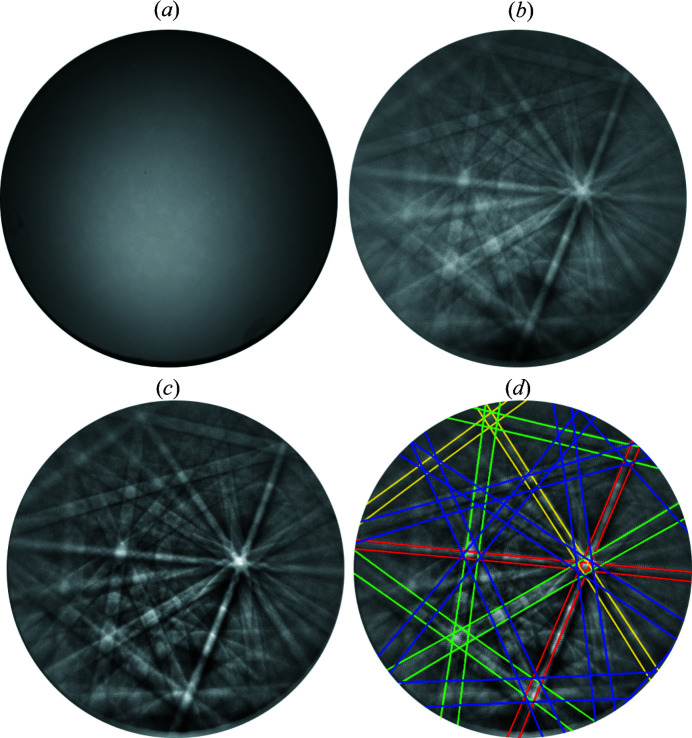
Treatment of BKD patterns. (*a*) BKD background collected from an Fe_40_Ni_40_B_20_ metallic glass, revealing some imperfections of the phospho­rus layer of the detector screen. (*b*) Martensite BKD pattern from Fe_21_Mn_58_Al_17_Ni_4_ alloy, as exported by the default settings of the acquisition program (*c*), externally corrected (*d*), tentatively indexed as f.c.c.: {111} in red, {200} in yellow, {220} in green and {311} in blue.

Special efforts were made to acquire BKD patterns with the best possible quality:

(i) The individual BKD patterns were acquired with an acquisition time of more than 100 ms. Ten patterns of the same spot were summed to reduce noise.

(ii) Background subtraction was done on the basis of BKD patterns acquired from embedded and polished Fe_40_Ni_40_B_20_ metallic glass having a similar electron density to the presently investigated alloy [see Fig. 2 ▸(*a*)]. This method is superior in the present case of a very coarse-grained alloy to averaging many differently oriented grains of the actual alloy, as the latter procedure leads to residual Kikuchi bands in the averaged background pattern dedicated to serve as background for the BKD indexing procedure.

(iii) The acquired BKD patterns were used in their 16-bit format and treated by low-pass filtering and adjusting of the gray value histogram [compare Fig. 2 ▸(*b*) and Fig. 2 ▸(*c*)] to reduce noise in the pattern, originating from an imperfect background subtraction.

The BKD patterns were analyzed by means of Hough-space-based indexing using the *TSL OIM DC 7* software (EDAX, 2017 ▸). In order to relate the experimentally measured patterns to possible structure models, dynamic simulation of the BKD patterns, using the structure models, was carried out using the Bruker *DynamicS* software (Winkelmann *et al.*, 2007 ▸).

## 3. Results and discussion   

### 3.1. Evaluation of the BKD patterns   

**Figure 3:**
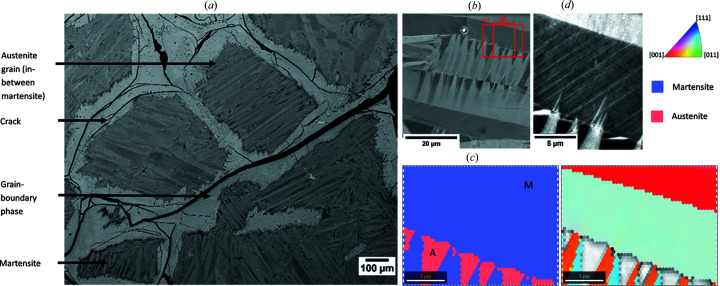
Microstructure of the investigated Fe_21_Mn_58_Al_17_Ni_4_ alloy. (*a*) SEM-BSE micrograph of the Fe_21_Mn_58_Al_17_Ni_4_ alloy quenched from 1473 K, depicting former austenite grains transformed during quenching (see text). The cracks were formed during sample preparation. (*b*) SEM-BSE micrograph [located in the middle of a grain in (*a*)] of the region of interest with areas (*c*) and (*d*) marked (red boxes). (*c*) EBSD phase map (left) and EBSD orientation map colored after cubic inverse pole figure coloring in the out-of-plane direction (right). The martensite is indexed using an f.c.c. structure (displayed in blue) and the austenite was indexed with a b.c.c. structure (red). Kikuchi patterns of austenite and martensite are marked with ‘A’ and ‘M’, respectively. (*d*) Electron channeling contrast imaging micrograph of a martensite lamella with visible linear contrasts.

The microstructures of the samples included in Fig. 1 ▸ always appear to be single-phased b.c.c./austenitic at the annealing temperature of 1473 K with grain sizes up to several hundred µm. Upon quenching, martensite was formed to an extent which is strongly composition dependent. In the case of the presently investigated Fe_21_Mn_58_Al_17_Ni_4_ alloy, starting from the grain boundaries, the β-manganese phase has formed, presumably during quenching [see Fig. 3 ▸(*b*)]. For more Fe-rich alloys there is the tendency to form a grain-boundary f.c.c. phase instead (see Vollmer *et al.*, 2015 ▸), which was avoided in the current work, however, by sufficiently rapid quenching.

The inner parts of the grains of the quenched Fe_21_Mn_58_Al_17_Ni_4_ alloy show pronounced varying backscattered electron (BSE) contrast [Fig. 3 ▸(*b*)]. These chemically homogeneous regions, as checked by EDS analysis, have obviously been transformed to martensite to a large extent, where the contrast variations are attributable to different orientations of the martensite variants in the size range of 10–100 µm. The BKD patterns taken from such regions can be tentatively indexed as either b.c.c. austenite (very small regions) or f.c.c. martensite [Fig. 3 ▸(*c*)]. Thereby, as expected, regions with homogeneous BSE contrast show a constant crystallographic orientation. However, electron channeling contrast imaging (ECCI) reveals that the regions of apparently homogeneous orientation, according to the tentative EBSD indexing, show fine-scale linear contrasts [Fig. 3 ▸(*c*) versus 3 ▸(*d*)], indicating some regular structural inhomogeneity (Zaefferer & Elhami, 2014 ▸) within the martensite variants.

**Figure 4:**
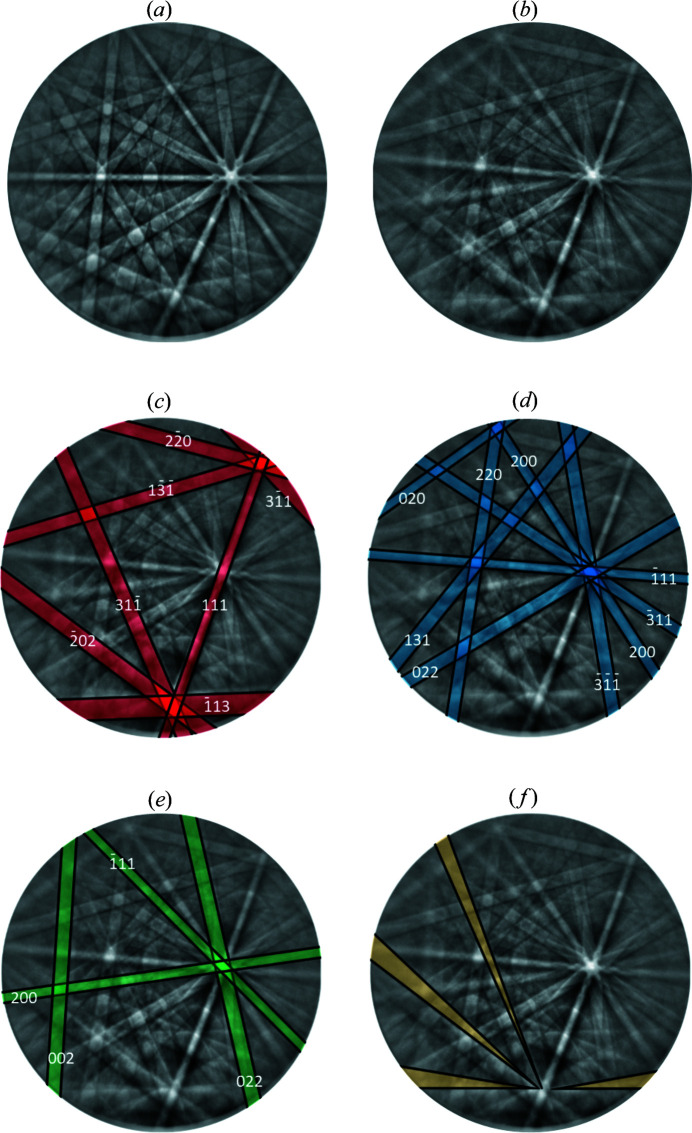
Kikuchi patterns taken from the quenched Fe_21_Mn_58_Al_17_Ni_4_ alloy. (*a*) Austenite BKD pattern taken from the region marked with ‘A’ in Fig. 3 ▸(*c*). (*b*) Martensite BKD pattern from the region marked with ‘M’ in Fig. 3 ▸(*c*). In the following figures, the martensite pattern is shown with indexed polytype-invariant bands (*c*), bands of the first (major) orientation (*d*) and the second (minor) orientation (*e*), and distortions in the shape of certain bands (*f*).

In order to analyze the origin of this structural heterogeneity in the martensite implied by the ECCI images, the BKD patterns were analyzed more closely. An acquired BKD pattern of the austenite phase is shown in Fig. 4 ▸(*a*). This was used to determine the pattern center. Fig. 4 ▸(*b*) shows a pattern taken from martensite [identical to Figs. 2 ▸(*b*)–2 ▸(*d*)]. Taking the tentative indexing suggested by the Hough-space-based method (*TSL OIM DC 7*), it becomes obvious that not all visible and detected bands are accounted for by the bands predicted for the assessed orientation [see Fig. 2 ▸(*d*)]. Note that these bands are narrow and hence cannot be bands with indices higher than those used for indexing in Fig. 2 ▸(*d*).

Kikuchi bands additional to those expected from the f.c.c. structure may be a sign of a more complicated, polytypic structure [as *e.g.* encountered by Omori *et al.* (2013 ▸)] or of twinning. To identify the polytype characteristics of the diffracting volume, bands due to the available f.c.c. indexing [Fig. 2 ▸(*d*)] are divided into bands which are (i) polytype invariant {for f.c.c. indexing *h* + *k* + *l* = 3*N* (Warren, 1990 ▸) with integer *N* taking [111] as the stacking direction, for hexagonal indexing *h* − *k* = 3*N* taking [001] as the stacking direction} and (ii) polytype specific (all others).

The polytype-invariant bands are shown in Fig. 4 ▸(*c*) beside the polytype-specific bands in Fig. 4 ▸(*d*). The band that stems from the lattice plane (111) is more pronounced than other bands stemming from the remaining equivalent lattice planes {111}. Thus, the stacking direction was assumed to be perpendicular to the lattice plane (111).

**Figure 5:**
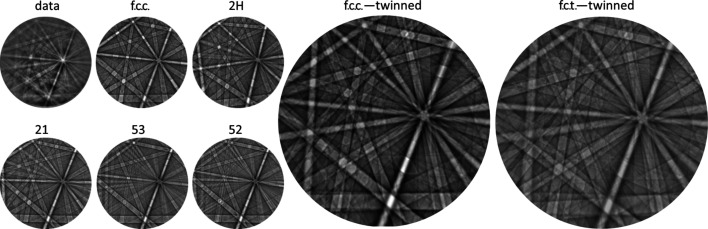
Measured BKD pattern (data), and dynamical simulation of different close-packed polytype structures labeled by their Zhdanov symbol. Moreover, superpositions of the dynamically simulated pattern of the f.c.c. structure (f.c.c. twin) and of a tetragonally distorted variant (f.c.t. twin, hence deviating from the close-packed structure principle; see text) are shown.

For the identified stacking direction [111], in Fig. 5 ▸ simulated Kikuchi patterns are shown for a series of different polytypes and of twinned f.c.c. structures. Obviously, the more complex polytypes 21, 53 and 52 produce Kikuchi patterns with polytype-specific bands that are significantly different from the experimentally observed ones [compare Fig. 4 ▸(*d*)]. Among the considered structures the simple Σ3 twin exhibits the best agreement with the experimental pattern. The second orientation introduced by such a Σ3 twin boundary within the (111) plane can be crystallographically described either by a rotation of the crystal lattice of 60° around the direction [111] or by a rotation of 70.5° around the direction 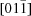. To achieve the simulated Σ3 twin pattern, the intensities of two dynamically simulated Kikuchi patterns of corresponding orientations were arithmetically averaged (see Fig. 5 ▸).^2^ That pattern accounts reasonably well for the polytype-specific bands [Fig. 4 ▸(*d*)] due to the Hough-based f.c.c. orientation, but also for the further bands that are otherwise unaccounted for [Fig. 4 ▸(*e*)]. These are the polytype-specific bands of the second (twin) orientation, whereas the polytype-invariant bands due to the second (twin) orientation should exactly overlap according to the simulated pattern. It appears that the bands due to the first orientation assigned by the Hough-based indexing [Fig. 2 ▸(*d*)] are more pronounced than those of the second orientation, implying differing volume fractions of the specific orientations within the excitation volume of the electron beam. Accordingly, these two orientations are referred to as the ‘majority’ and ‘minority’ orientations.

Analysis of the experimental BKD pattern in terms of mutual presence of f.c.c. martensite with a majority and a minority orientation has been done separately within the *TSL OIM DC 7* software. First, only the automatically detected Hough peaks due to the polytype-invariant bands as well as the (strong) polytype-specific bands due to majority orientation were chosen as input to determine the majority f.c.c. orientation. In order to enforce indexing based on the remaining weaker bands of the minority orientation, band orientations were indicated manually within the *TSL OIM DC 7* software based on the experimental pattern. These bands could be indexed with a different, minority f.c.c. orientation. The orientation relationship between majority and minority orientations thus determined agrees well with the (Σ3) twin orientation relationship.

The co-existence of two different orientations in the excitation volume of the electron beam is the reason for the varying quality of f.c.c. indexing, as the acquisition program uses a Hough-based algorithm to index the BKD patterns. For this, a distinct number of bands is detected, starting with the band of the highest intensity (Winkelmann *et al.*, 2007 ▸). For patterns with good pattern quality, nearly all bands used originate from the same (major) orientation because enough bands are detected. For poor pattern quality, most of the broad bands are not detected, causing the detected bands to be a mixture of just the bands with low *hkl* values of both diffracting orientations. This also explains the high success rate of patterns indexed with one single f.c.c. orientation in the case of good pattern quality and a martensite orientation showing a low number of minor bands.

Nevertheless, there exist some minor discrepancies between the experimental pattern and the simulated f.c.c. twin. As highlighted in Fig. 4 ▸(*f*), the polytype-invariant bands show triangular features, implying that the polytype-invariant bands that originate from the major and minor orientations do not coincide entirely. Since this splitting of the bands is not observed for the simulated patterns due to the f.c.c. twin, this model does not completely account for the observed diffraction patterns. A typical observed feature in martensites, which can also account for the splitting of the observed bands, is the tetragonal distortion of the unit cell. Tetragonality is a common type of distortion encountered for many martensite crystal structures resulting from a b.c.c. → f.c.c. or f.c.c. → b.c.c. transformation for various reasons (see *e.g.* Christian, 1992 ▸) and, hence, this is an obvious possibility.

**Figure 6:**
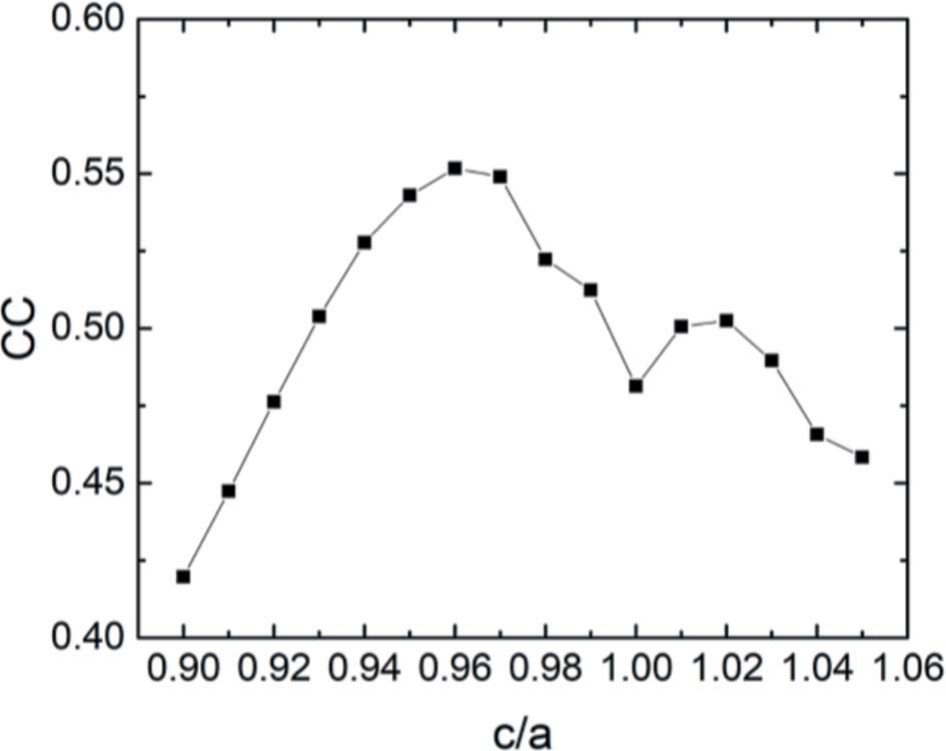
Cross-correlation coefficient of the dynamically simulated BKD pattern in comparison with the measured pattern for different *c*/*a* ratios. The simulation was performed only for the major orientation of the pattern. The maximum CC indicates the best agreement.

To assess such a tetragonal distortion of the diffracting crystal structure, the experimental BKD pattern in Fig. 4 ▸(*b*) was compared with different dynamical simulations of tetragonally distorted f.c.c. (f.c.t.) structure^3^ obtained using the Bruker *DynamicS* program (Winkelmann *et al.*, 2007 ▸). To determine the tetragonality, the simulated patterns were compared with the experimental pattern while refining the orientation for the simulated patterns upon maximizing the cross-correlation coefficient (CC) (Britton & Hickey, 2018 ▸). To keep the procedure simple, only the major orientation has been used for the simulations. As the absolute lattice parameters only affect the band widths of the simulated BKD pattern, the important parameter to vary is the ratio of the tetragonal lattice parameters *c*/*a* (referring to a face-centered tetragonal unit cell). Fig. 6 ▸ shows the maximized CC for the different tested *c*/*a* ratios. The best agreement was found for *c*/*a* = 0.96.

Comparison of experimental data and the superposition of the dynamically simulated f.c.t. structure shows qualitatively a good fit. Since the majority variant was assumed to be tetragonal, it was taken as likely that the minority variant also shows the same tetragonal distortion. As the *DynamicS* program was used to analyze the tetragonal distortion, evaluation based on manually indicated bands was continued using this software, equivalently to what has been described above for the f.c.c. structure: the bands due to the majority orientation were selected on the basis of the automatically detected Hough peaks, those due to the minority orientation were selected manually. (Use of the cross-correlation coefficient for the described purpose was not feasible because it was not possible to enforce indexing of the minority component in this way without falling into the deeper minimum due to the bands of the majority orientation.)

**Figure 7:**
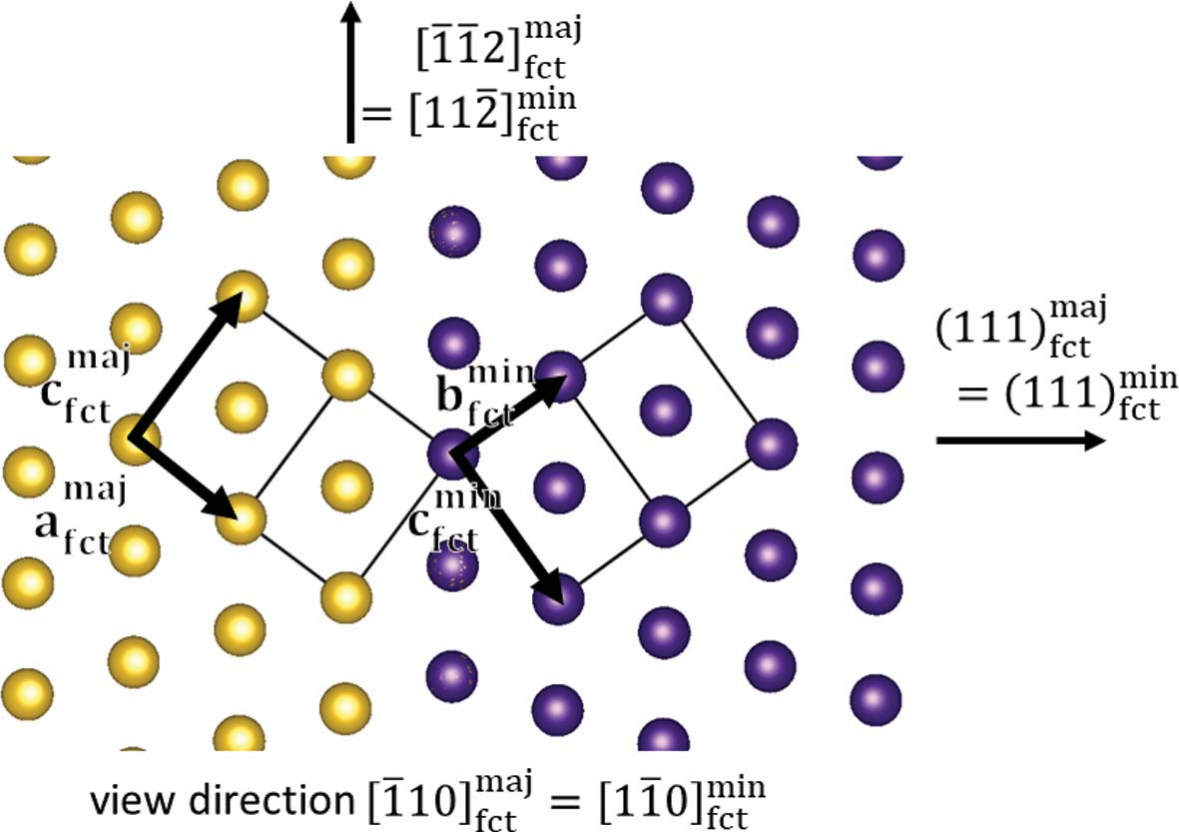
Schematic representation of the f.c.t. twin with the vertical (111) twinning plane shown along the common 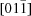 direction.

**Figure 8:**
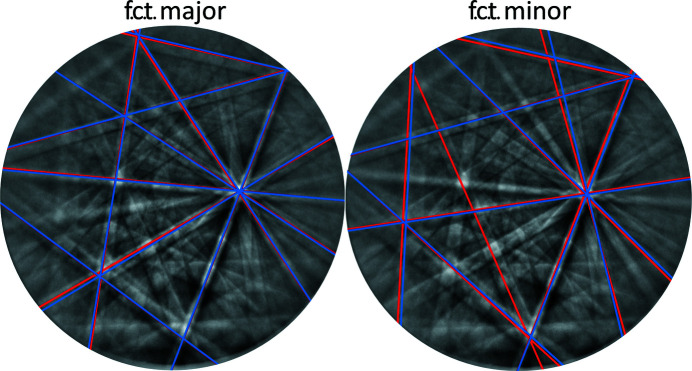
Input bands (red) used for the determination of the major and minor f.c.t. orientation in the *DynamicS* software. The blue bands are the respective simulated bands for the orientation.

The major and minor orientations obtained from indexing imply the following orientation relationship: 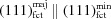 and 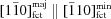 or 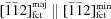 (Fig. 7 ▸), as well as 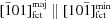 and 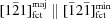 *etc*., with maj and min referring to majority and minority, respectively. The experimentally observed orientation determined in the described way deviates only by 0.21° from this ideal orientation. The quality of the orientation determination using the *DynamicS* software is visible in Fig. 8 ▸. The red bands in this figure indicate the input bands for the orientation determination and the blue bands indicate the respective bands of the fitted orientation. The blue bands seem to correspond very well with the underlying measured pattern. A detailed analysis of the fit of the simulated structures with the measured BKD pattern is shown in Appendix *A* [App appa].

**Figure 9:**
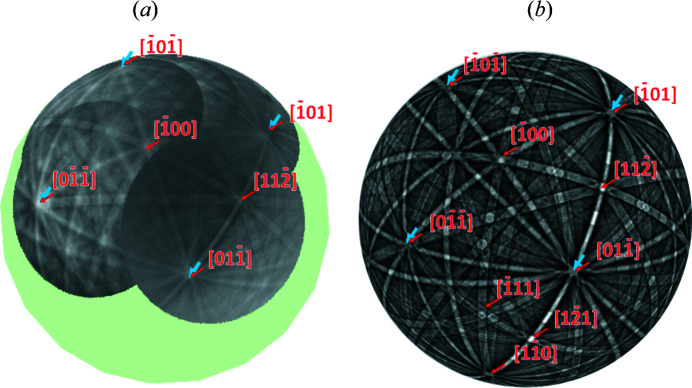
Comparison of experimental data with simulated patterns. (*a*) Measured BKD pattern of the same martensite grain, generated by rotation of the sample. The green sphere indicates the theoretical size of the BKD sphere. (*b*) Superposition of dynamically simulated BKD spheres for a twinned f.c.t. martensite. Zone axes are numbered and colored according to the measured pattern. The indexing is with respect to the major orientation.

The orientation relationship ensures the absence of misfit and hence full coherency, if the 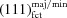 planes are also habit planes. The parallelism of some of the mentioned directions in Fig. 4 ▸ is further emphasized in Fig. 9 ▸, which reconstructs a larger part of the Kikuchi sphere on the basis of a series of patterns obtained for different rotation angles of the sample around its surface normal in the vicinity of the electron beam. By using this procedure (Fischer *et al.*, 2019 ▸) it is possible to show a larger number of zone axes of a BKD sphere with a low symmetry, maintaining nearly the same diffracting volume. With this procedure it can be shown that the twinned f.c.t. structure describes not only the hitherto shown BKD pattern from Fig. 4 ▸ very well but also a large part of the BKD sphere [compare measured data in Fig. 9 ▸(*a*) and dynamical simulation in Fig. 9 ▸(*b*)]. In Fig. 9 ▸ the corresponding 〈110〉 zone axis is marked in blue.

### 3.2. Implications for f.c.c. martensite in Fe–Mn–Al–Ni alloys   

The present results imply that, for the investigated alloy composition, thermally generated martensite is a tetragonally distorted and twinned f.c.c. polytype. Tentative analysis of Kikuchi patterns of other compositions from thermally generated martensite implies the presence of these structural features over a large range of composition (see Fig. 1 ▸). The results presented here for the structure of the martensite, however, differ from the 53 polytype deduced by Omori *et al.* (2012 ▸) based on selected-area electron diffraction of martensite generated in Fe_43.5_Mn_34_Al_15_Ni_7.5_ and from the apparently single-orientation f.c.c. structure as reported by Vallejos *et al.* (2018 ▸).

Both the short-range periodic faulting present in a 53 polytype and the present type of twinning definitely correspond to a mode of lattice-invariant shear, ensuring that martensite forms by an invariant plane strain (Zhang & Kelly, 2009 ▸). This indicates that the mode of lattice-invariant shear may vary in this type of alloy. As it concerns the observed tetragonality, in the field of martensites not containing interstitials, this is frequently a consequence of inherited order from the austenitic state (Christian, 1992 ▸). All this insight should be included in predictions of crystallographic and microstructural features (like habit planes) of the martensites in Fe–Mn–Al–Ni alloys.

## 4. Summary and conclusion   

Detailed evaluation of backscatter Kikuchi diffraction patterns from f.c.c.-like martensite, which had formed upon quenching of b.c.c.-Fe_21_Mn_58_Al_17_Ni_4_ austenite, has been carried out. The results can be summarized as follows:

(i) Although tentative Hough-space indexing succeeded using an f.c.c. structure model, analysis of the patterns revealed Kikuchi bands from the independently diffracting f.c.c. minority orientation, which coexists with the majority orientation in the diffracting volume.

(ii) More detailed analysis of the Kikuchi band positions revealed, for both orientations, a tetragonal distortion with an approximate axial ratio of 0.96 (referring to a face-centered tetragonal cell), as determined by cross-correlation analysis in comparison with dynamically simulated patterns.

(iii) The two orientations assume a twin relationship 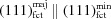 and 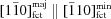, with maj and min referring to the majority and minority orientations, respectively, which allows with 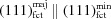 contact planes a coherent twin interface.

(iv) The observed twinning is most likely the mode of lattice-invariant shear ensuring an invariant plane strain in the course of the martensite formation.

Thereby, it is demonstrated that the detailed band analysis of BKD patterns can reveal structure distortions and independently diffracting orientations or phases within the excitation volume. Such information is typically obtained by transmission electron microscopy (TEM) supported by selected-area electron diffraction; however, this requires much more sophisticated preparation, to obtain electron-transparent specimens. The current procedure can be conducted on conventionally prepared metallographic specimens, where it is possible to investigate significantly larger specimen areas compared with TEM. In cases where the preparation of appropriate TEM specimens is possible, this technique could be used complementarily, since TEM still has important merits, *e.g.* in revealing weak reflections not detectable as Kikuchi bands or in detecting diffuse scattering and important real-space information like defects and habit planes.

## Figures and Tables

**Figure 10:**
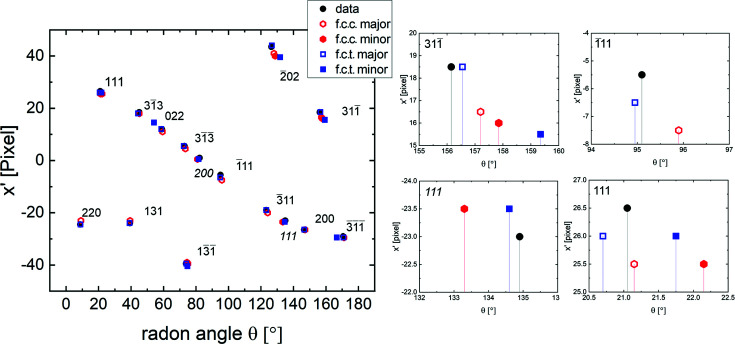
Location of maxima of the Radon transform of the measured data and different dynamically simulated BKD patterns. The distance from the center of the pattern (not the pattern center) is plotted against the angle for the bands with the strongest intensities. Bands are annotated and multiple magnifications of different bands are shown, to give insight into the quality of the fit of the different dynamically simulated structures.

## Footnotes

1 Indicating five lattice planes with ascending stacking sequence, followed by three lattice planes with descending stacking sequence (*e.g.* *ABCABACB*).

2 A corresponding linear combination of such patterns is justified if the regions of the two orientations diffract incoherently. However, even an ideal stack of close-packed layers with f.c.c. structures exhibiting two twin orientations will approximate to such a pattern if the twin domains are sufficiently large. Small domains of random size will mainly lead to streaking/broadening in a diffraction pattern, which is, however, not easily discernible in a BKD pattern of the present type. The term ‘nanotwinned’ applied to the polytypes, *e.g.* to the 53 polytype in the work of Omori *et al.* (2012 ▸), is misleading insofar as the diffraction pattern is that of a corresponding polytype and differs considerably from a twin with independently diffracting twin regions.

3 Note that the standard crystallographic description of a face-centered tetragonal structure, *i.e.* a close-packed cubic arrangement of atoms having experienced some tetragonal distortion, occurs in *I*4/*mmm* symmetry, *i.e.* using a body-centered tetragonal Bravais lattice (Aroyo, 2016 ▸). In the present work, however, the lattice parameters refer to the occasionally employed enlarged unit cell implying an *F*4/*mmm* description of the *I*4/*mmm* space group.

## Appendix A

To quantitatively evaluate the accuracy of the model crystal structure in describing the measured data, the measured BKD pattern and simulations are subjected to a Radon transformation (Britton *et al.*, 2018 ▸). Fig. 10 ▸ shows the position of selected bands in the Radon space, including magnifications of different bands. Relevant is the angular position θ measured around the center of the pattern. Bands of the minor phase are indexed and shown in italics. The f.c.t. twin is separated into the major and minor orientations. The Radon space provides the opportunity to evaluate the angular deviation between measured data and simulation for each band separately. From the figure it can be seen that the f.c.t. orientations result in the best fit for the band positions. The differences in θ between the measured data and the f.c.t. crystal structures can be ascribed to the accuracy of the Radon transformation.

## References

[bb1] Aroyo, M. J. (2016). Editor. *International Tables for Crystallography*, Vol. A, *Space-Group Symmetry*. Chichester: Wiley.

[bb2] Britton, T. B. & Hickey, J. L. R. (2018). *IOP Conf. Ser. Mater. Sci. Eng.* **304**, 12003.

[bb3] Britton, T. B., Tong, V. S., Hickey, J., Foden, A. & Wilkinson, A. J. (2018). *J. Appl. Cryst.* **51**, 1525–1534.

[bb4] Christian, J. W. (1992). *Mater. Trans. JIM*, **33**, 208–214.

[bb30] EDAX (2017). *TSL OIM Analysis V7*. EDAX, Mahwah, NJ, USA.

[bb5] Fischer, P., Rudolph, M. & Leineweber, A. (2019). *An Experimental Code For the Partial Reconstruction Of a Kikuchi Sphere From Recorded EBSD Patterns*, https://github.com/Martin-Rudolph/Kikuchi.

[bb6] La Roca, P., Medina, J., Sobrero, C. E., Avalos, M., Malarria, J. A., Baruj, A., Sade, M., Schryvers, N. & van Humbeeck, J. (2015). *MATEC Web Conf.* **33**, 4005.

[bb7] Omori, T., Ando, K., Okano, M., Xu, X., Tanaka, Y., Ohnuma, I., Kainuma, R. & Ishida, K. (2011). *Science*, **333**, 68–71. 10.1126/science.1202232 21719673

[bb8] Omori, T., Nagasako, M., Okano, M., Endo, K. & Kainuma, R. (2012). *Appl. Phys. Lett.* **101**, 231907.

[bb9] Omori, T., Okano, M. & Kainuma, R. (2013). *APL Mater.* **1**, 32103.

[bb10] Tseng, L. W., Ma, J., Hornbuckle, B. C., Karaman, I., Thompson, G. B., Luo, Z. P. & Chumlyakov, Y. I. (2015*a*). *Acta Mater.* **97**, 234–244.

[bb11] Tseng, L. W., Ma, J., Vollmer, M., Krooß, P., Niendorf, T. & Karaman, I. (2016*a*). *Scr. Mater.* **125**, 68–72.

[bb12] Tseng, L. W., Ma, J., Wang, S. J., Karaman, I. & Chumlyakov, Y. I. (2016*b*). *Scr. Mater.* **116**, 147–151.

[bb13] Tseng, L. W., Ma, J., Wang, S. J., Karaman, I., Kaya, M., Luo, Z. P. & Chumlyakov, Y. I. (2015*b*). *Acta Mater.* **89**, 374–383.

[bb14] Vallejos, J. M., Sobrero, C. E., Ávalos, M., Signorelli, J. W. & Malarría, J. A. (2018). *J. Appl. Cryst.* **51**, 990–997.

[bb15] Vollmer, M., Kriegel, M. J., Walnsch, A., Klemm, V., Leineweber, A. & Niendorf, T. (2019). *Scr. Mater.* **162**, 442–446.

[bb16] Vollmer, M., Krooß, P., Karaman, I. & Niendorf, T. (2017). *Scr. Mater.* **126**, 20–23.

[bb17] Vollmer, M., Krooß, P., Kriegel, M. J., Klemm, V., Somsen, C., Ozcan, H., Karaman, I., Weidner, A., Rafaja, D., Biermann, H. & Niendorf, T. (2016). *Scr. Mater.* **114**, 156–160.

[bb18] Vollmer, M., Segel, C., Krooß, P., Günther, J., Tseng, L. W., Karaman, I., Weidner, A., Biermann, H. & Niendorf, T. (2015). *Scr. Mater.* **108**, 23–26.

[bb19] Walnsch, A., Kriegel, M. J., Fabrichnaya, O. & Leineweber, A. (2019*a*). *Calphad*, **66**, 101621.

[bb20] Walnsch, A., Kriegel, M. J., Rudolph, M., Motylenko, M., Fabrichnaya, O. & Leineweber, A. (2019*b*). *Calphad*, **64**, 78–89.

[bb21] Warren, B. E. (1990). *X-ray Diffraction*. New York: Dover.

[bb22] Winkelmann, A., Trager-Cowan, C., Sweeney, F., Day, A. P. & Parbrook, P. (2007). *Ultramicroscopy*, **107**, 414–421. 10.1016/j.ultramic.2006.10.006 17126489

[bb23] Zaefferer, S. & Elhami, N.-N. (2014). *Acta Mater.* **75**, 20–50.

[bb24] Zhang, M.-X. & Kelly, P. M. (2009). *Prog. Mater. Sci.* **54**, 1101–1170.

[bb25] Zhdanov, G. S. (1945). *C. R. Acad. Sci. URSS*, **48**, 36–42.

